# Neutralizing activity of intravenous immune globulin products against enterovirus D68 strains isolated in Japan

**DOI:** 10.1186/s12879-023-08429-z

**Published:** 2023-07-18

**Authors:** Kazuhiro Yoshida, Masamichi Muramatsu, Hiroyuki Shimizu

**Affiliations:** 1 Department of Virology 2, National Institute of Infectious Diseases, Tokyo, Japan; 2 Department of Infectious Disease Research, Institute of Biomedical Research and Innovation, Foundation for Biomedical Research and Innovation at Kobe, Kobe, Hyogo Japan

**Keywords:** IVIG, Enterovirus D68, Neutralization activity, Japan

## Abstract

**Background:**

Enterovirus D68 (EV-D68), belonging to *Enterovirus D*, is a unique human enterovirus mainly associated with common respiratory diseases. However, EV-D68 can cause severe respiratory diseases, and EV-D68 endemic is epidemiologically linked to current global epidemic of acute flaccid myelitis.

**Methods:**

In this study, we measured neutralizing antibody titers against six clinical EV-D68 isolates in nine intravenous immune globulin (IVIG) products commercially available in Japan to assess their potential as therapeutic options for severe EV-D68 infection.

**Results:**

Seven IVIG products manufactured from Japanese donors contained high neutralizing antibody titers (IC_50_ = 0.22–85.01 µg/mL) against all six EV-D68 strains. Apparent differences in neutralizing titers among the six EV-D68 strains were observed for all IVIG products derived from Japanese and non-Japanese blood donors.

**Conclusions:**

High levels of EV-D68–neutralizing antibodies in IVIG products manufactured from Japanese donors suggest that anti-EV-D68 antibodies are maintained in the Japanese donor population similarly as found in foreign blood donors. Apparent differences in neutralizing antibody titers against the six EV-D68 strains suggest distinct antigenicity among the strains used in this study regardless of the genetic similarity of EV-D68.

## Background

Human enteroviruses are members of the *Picornaviridae* family, and they are categorized into four species (A–D). They are non-enveloped viruses with positive single-stranded RNA genome. Enteroviruses cause various clinical symptoms, including hand, foot, and mouth disease, herpangina, aseptic meningitis, poliomyelitis, acute encephalitis, acute flaccid paralysis (AFP), and acute flaccid myelitis (AFM). The fecal–oral and respiratory routes are the major infectious routes of enteroviruses [1].

Enterovirus D68 (EV-D68) was initially isolated from children with pneumonia and bronchiolitis in California in 1962 [2], and it belongs to *Enterovirus D*. Currently, clades A*–*D are identified as the circulating subtypes of EV-D68 [3, 4], and clades A, B, and C are predominantly detected in Japan [5, 6]. Some of the virological characteristics of EV-D68 are similar to those of human rhinoviruses, which belong to *Rhinovirus A–C* within the genus *Enterovirus*, and the main symptoms are nasal mucus, cough, asthma-like attacks, and pneumonia [3, 4, 7]. However, in rare cases, EV-D68 can cause AFM, a polio-like neurological disease with an acute onset of flaccid limb weakness and spinal cord gray matter lesions on magnetic resonance imaging [8–12]. Previous reports indicated that EV-D68 was associated with the onset of AFM, as an EV-D68 epidemic was reported at same time when an increase in AFM cases was occurred [13–15].

From 1970 to 2005, 26 cases of EV-D68 infection were reported to the National Enterovirus Surveillance System in the United States [16], and after 2005, small clusters of EV-D68 epidemics occurred in North America, Asia, and Europe [17, 18]. In 2014, a large outbreak of EV-D68 was reported in the United States, and 1,395 confirmed cases of respiratory illness were recorded from August 2014 to January 2015 by the Centers for Disease Control and Prevention [13, 19–21].

In Japan, an EV-D68 outbreak occurred from August to December in 2015 [22], and a clade B strain was detected around the time of the outbreak [23, 24]. Additionally, the presence of clades A–C strains has expanded in recent years [5, 6, 25].

To treat EV-D68 infections, the anti-enterovirus activity of intravenous immune globulin (IVIG) products has been examined in vitro and in vivo. A previous report found that five IVIG products used in the United States and Europe have neutralizing activity against three EV-D68 strains derived from outbreaks in 2014 in Missouri, Illinois, and Kentucky [26]. In mice infected with an EV-D68 strain, IVIG products reduced paralysis symptoms and decreased spinal cord viral loads [27]. Additionally, monoclonal antibodies with neutralizing activity against an EV-D68 strain derived from Missouri in 2014 also reduced paralysis after paralysis onset in a mouse model with AFM caused by EV-D68 [28].

Currently, no vaccine against EV-D68 infection has been developed, and no therapeutic agents are available. Therefore, as the most realistic therapeutic option, IVIG products with neutralizing activity against EV-D68 strains will be used clinically. However, whether the IVIG products used in Japan contain neutralizing antibodies against domestic EV-D68 strains has not been clarified.

In this study, we examined whether IVIG products used in Japan have neutralizing activity against domestic EV-D68 strains. We used the EV-D68 strains isolated in Yamagata prefecture, Japan in 2010–2015.

## Methods

### Cell line

RD-A cells, a variant of the RD cell line (derived from human rhabdomyosarcoma), were gifted from the Centers for Disease Control and Prevention in United States, and were maintained in Eagle’s Minimum Essential Medium (EMEM) (M4655; Sigma) supplemented with 10% fetal calf serum (FCS), 100 U/mL penicillin, and 100 µg/mL streptomycin (growth medium) or EMEM supplemented with 2% FCS, 100 U/mL penicillin, and 100 µg/mL streptomycin (maintenance medium). RD-A cells were passaged with growth medium once per week, and the medium was changed to maintenance medium 2 days after passage.

### IVIG products

Nine IVIG products were obtained from five manufacturers in Japan and one each in the United States and Germany (Table 1). Seven products were produced using donated blood in Japan, the product manufactured in Germany was also produced using donated blood, and the product manufactured in the United States was derived from non-donated blood.

**Table 1:** IVIG products used for the EV-D68 neutralization test

IVIG product	Manufacturer	IVIG concentration (mg/mL)	Form of antibodies	Chemical modification	Blood donor	
					Country	Source
Product 1	A	50	Complete	Sulfonation	Japan	Donated blood
Product 2	B	50	Complete	–^a^	Japan	Donated blood
Product 3	C	50	Complete	–	Japan	Donated blood
Product 4	C	100	Complete	–	Japan	Donated blood
Product 5	D	50	Incomplete	–	Japan	Donated blood
Product 6	E	50	Complete	–	Japan	Donated blood
Product 7	E	100	Complete	–	Japan	Donated blood
Product 8	F	50	Complete	–	Germany	Donated blood
Product 9	G	50	Complete	–	USA	Non-donated blood

^a^ There are no chemical modification.

### EV-D68 strains

Six EV-D68 strains were isolated from patients with acute respiratory symptoms in Yamagata prefecture, Japan in 2010–2015 [5, 6]. The strains were classified to three distinct EV-D68 genetic clades. The clade A strains were 2076-Yamagata-2010 (National Center for Biotechnology Information accession No. AB614440) and 2006-Yamagata-2013 (LC203537), the clade B strains were 1975-Yamagata-2010 (AB614409) and 1576-Yamagata-2015 (LC203542), and the clade C strains were 2150-Yamagata-2010 (AB614419) and 2192-Yamagata-2010 (AB614422) (Table 2). The 50% cell culture infectious dose (CCID_50_) of each strain was calculated by the Kärber formula [29].

**Table 2:** EV-D68 strains used as the challenge virus

EV-D68 strain	Year of isolation	EV-D68 genogroup	Reference	
			Accession No.	Reference
2076-Yamagata	2010	Clade A/lineage 3^a^	AB614440	5
2006-Yamagata	2013	Clade A/lineage 3	LC203537	6
1975-Yamagata	2010	Clade B/lineage 2	AB614409	5
1576-Yamagata	2015	Clade B/lineage 2	LC203542	6
2150-Yamagata	2010	Clade C/lineage 1	AB614419	5
2192-Yamagata	2010	Clade C/lineage 1	AB614422	5

^a^ Each lineage is a genetically monophyletic group, similarly as a clade. We used the classification of “lineage” and “clade” categorized in the Ikeda et al. and Itagaki et al. reports [5, 6].

### Neutralization assay

We performed a neutralization assay once as described previously for human parechoviruses with minor modifications [30]. RD-A cells (1.5 × 10^5^ cells/mL) were seeded in 96-well plates, and 10 wells per dilution and per IVIG product were prepared. The next day, 4-fold serial dilutions of the IVIG products were added to growth medium containing each EV-D68 strain (final concentration, 100 CCID_50_/200 µL). The medium mixed with IVIG and EV-D68 was incubated for 1 h at 37 °C, and the supernatant of cultured RD-A cells was replaced with 200 µL/well mixed medium. RD-A cells were incubated in the mixed medium for 6 days at 35 °C, and cytopathic effects (CPEs) were observed. Additionally, we confirmed whether RD-A cells infected with each EV-D68 strain in all wells exhibited apparent CPE as the 100 CCID_50_/200 µL condition except that no IVIG product was added. The 50% inhibitory concentration (IC_50_), which was the concentration of each IVIG product that neutralized EV-D68 by 50%, was calculated from the observation of CPEs using the Kärber formula with positive wells as neutralized ones. Ten wells per dilution and per IVIG product were used for calculation of IC_50_.

## Results

### Nine IVIG products neutralized the EV-D68 strains isolated in Japan

**Fig. 1:**
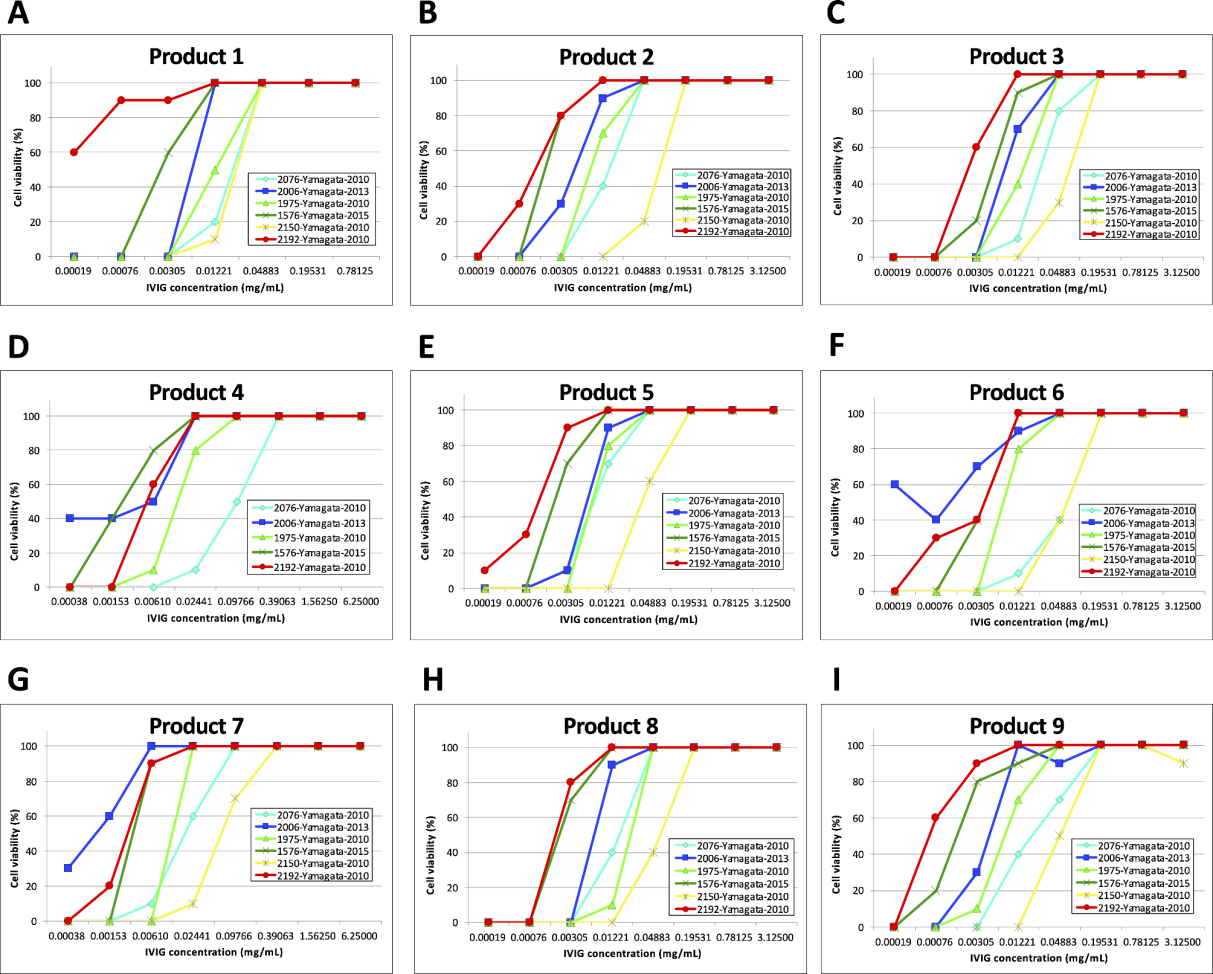
Neutralization of the EV-D68 strains by IVIG products. The neutralizing activity was performed once using RD-A cells (1.5 × 10^5^ cells/mL) seeded in 96-well plates, and 10 wells per dilution and per IVIG product were prepared. On the next day, 4-fold serial dilutions of the IVIG products were added to growth medium containing each EV-D68 strain (final concentration, 100 CCID_50_/200 µL). The medium containing IVIG and EV-D68 was incubated for 1 h at 37 °C, and the supernatant of cultured RD-A cells was replaced with 200 µL/well mixed medium. RD-A cells in the mixed medium were incubated for 6 days at 35 °C, and CPEs were observed. **A**–**I** represents products 1–9, respectively. Diamond, 2192-Yamagata-2010; square, 1975-Yamagata-2010; triangle; 2006-Yamagata-2013; cross, 1576-Yamagata-2015; asterisk. 2150-Yamagata-2010; circle, 2076-Yamagata-2010. The apparent CPEs were observed in all assayed wells when the product 1 was used as the highest concentration (3.125 mg/mL). The back titration of 2076, 2006, 1975, 1576, 2150, and 2192 EV-D68 strains were 66.99 ± 51.08, 33.66 ± 32.54, 41.61 ± 25.91, 108.36 ± 87.86, 66.49 ± 29.56, and 24.93 ± 23.52 CCID_50_/200 µL, respectively, and the CPEs were observed in all wells under 100 CCID_50_/200 µL condition without each IVIG product

We performed a neutralization assay to examine whether IVIG products used in Japan have neutralizing activity against EV-D68 strains (Fig. 1). Using RD-A cells infected with EVs, all nine IVIG products neutralized EV-D68 strains isolated in Japan, which included three lineages (clades A–C). The back titration of 2076, 2006, 1975, 1576, 2150, and 2192 EV-D68 strains were 66.99 ± 51.08, 33.66 ± 32.54, 41.61 ± 25.91, 108.36 ± 87.86, 66.49 ± 29.56, and 24.93 ± 23.52 CCID_50_/200 µL, respectively, and the CPEs were observed in all wells under 100 CCID_50_/200 µL condition without each IVIG product. Apparent differences in neutralizing antibody titers against the six EV-D68 strains were observed for the IVIG products derived from Japanese and foreign blood donors (see the next section), suggesting distinct antigenicity among the strains used in this study regardless of the genetic similarity of EV-D68. Although the two clade C strains (2150-Yamagata-2010 and 2192-Yamagata-2010) were closely related phylogenetically, the neutralizing activities of the IVIG products were apparently different. The neutralization assay indicated that IVIG products used in Japan can neutralize EV-D68 strains prevalent in Japan.

### IC_50_ determination

We calculated the IC_50_ from the CPEs observed in the neutralization assay (Table 3). The IC_50_ values of the IVIG products ranged 0.22–85.01 µg/mL. The mean IC_50_ values of products 1–9 as listed in Table 3 against the six EV-D68 strains were 10.16, 17.54, 20.62, 21.74, 11.34, 19.91, 17.03, 17.12, and 15.42 µg/mL, respectively. The seven IVIG products manufactured from Japanese donors contained neutralizing antibodies (IC_50_ = 0.22–85.01 µg/mL) against the six EV-D68 strains. Additionally, we calculated the difference of the IC_50_ between the two strains in each clade (Table 4). The average differences were 23.89 ± 25.76, 9.05 ± 4.29, and 52.74 ± 15.59 µg/mL in clades A, B, and C, respectively.

**Table 3:** IC_50_ in the neutralization assay^a, b^

Product	EV-D68 strain					
	2076	2006	1975	1576	2150	2192
Product 1	18.50^c^	6.10	12.21	2.66	21.25	0.22
Product 2	14.02	4.63	9.25	2.01	74.01	1.33
Product 3	28.04	9.25	14.02	5.31	64.43	2.66
Product 4	85.01	2.01	14.02	2.31	–^d^	5.31
Product 5	8.05	6.10	8.05	2.31	42.51	1.01
Product 6	48.83	0.66	8.05	3.51	56.09	2.31
Product 7	18.50	0.88	12.21	3.51	64.43	2.66
Product 8	14.02	7.01	21.25	2.31	56.09	2.01
Product 9	21.25	4.63	8.05	1.75	56.09	0.76

^a^ The unit of the IC_50_ is µg/mL.

^b^ IC_50_ was calculated from the Kärber formula with positive wells as neutralized ones, and 10 wells per dilution and per IVIG product were used for calculation of IC_50_.

^c^ We performed the neutralization assay once, therefore the standard deviation is not available.

^d^ This IC_50_ was not available.

**Table 4:** Difference of the IC_50_ in each clade^a^

Product	EV-D68 clade		
	Clade A	Clade B	Clade C
Product 1	12.40	9.55	21.03
Product 2	9.40	7.24	72.68
Product 3	18.79	8.71	61.77
Product 4	83.00	11.71	–^b^
Product 5	1.95	5.74	41.50
Product 6	48.16	4.55	53.78
Product 7	17.63	8.70	61.77
Product 8	7.01	18.94	54.08
Product 9	16.63	6.30	55.33

^a^ The difference of the IC_50_ (µg/mL) was calculated between 2076-Yamagata-2010 and 2006-Yamagata-2013 (clade A), between 1975-Yamagata-2010 and 1576-Yamagata-2015 (clade B), and between 2150-Yamagata-2010 and 2192-Yamagata-2010 (clade C).

^b^ The IC_50_ of the 2150 strain (product 4) was not available, therefore this difference was not calculated.

## Discussion

In this study, we found that all nine IVIG products used in Japan neutralized six EV-D68 strains isolated in Yamagata prefecture Japan in 2010–2015, which were categorized into three clades (clades A–C). Additionally, apparent differences in neutralizing titers among the six strains were observed for all IVIG products, and although the two clade C strains (2150-Yamagata-2010 and 2192-Yamagata-2010) were phylogenetically related [5, 6], different neutralizing titers were measured for each IVIG product, suggesting that the antigenicity among the strains used in this study was not related to the genetic similarity of EV-D68.

A previous report indicated that antisera against clade A–C stains have neutralizing activity only against strains in the same clade as the antigen used to obtain the antisera [31], whereas the IVIG products used in this study exhibited neutralizing activity against strains from clades A–C. This was because all of the IVIG products used in this study contained antibodies against clade A–C strains, and the extent of the neutralizing activity against the EV-D68 strains was different for each IVIG product.

Although molecular detection and identification are currently common for the laboratory diagnosis of EV-D68 infection, conventional virus isolation approaches might permit identification of the predominant EV-D68 strains for neutralization assays and seroprevalence studies [5, 6].

Vaccines and therapeutic agents against EV-D68 infection have not been introduced. Therefore, IVIG products represent a realistic and feasible option for the treatment of severe EV-D68–associated diseases. Whereas differences in neutralizing activity against different strains were revealed for the IVIG products investigated in this study, all IVIG products contained neutralizing antibodies; therefore, they are expected to have therapeutic efficacy against the three examined EV-D68 lineages. From the experience with polio and EV-A71 vaccines, there is a consensus regarding the importance of serum neutralizing antibodies for the prevention of clinical symptoms during the course of polio/enterovirus infections [32, 33]. Although previous research observed effective neutralizing antibodies against EV-D68 in animal experiments, the clinical efficacy of IVIG in humans remains unconfirmed [27, 28]. AFM has been identified as an EV-D68–neurological disease [34–40], and the possibility of anti-EV-D68 antibodies as preventive and therapeutic options for AFM was reported [41]. In Japan, a relationship between EV-D68 and AFM was suggested [12]. Therefore, IVIG products are expected to emerge as preventive or therapeutic options.

Seven IVIG products manufactured in Japan, in addition to products manufactured in Germany and the United States, exhibited neutralizing activity against EV-D68 in this study, indicating that the Japanese donor population maintains anti-EV-D68 antibodies similarly as foreign donors. In a recent seroepidemiological study, neutralizing antibodies were detected in individuals in the United States [42], Netherlands [43], China [44, 45], Malaysia [46], and Taiwan [47]. These findings were consistent with those of the present study revealing high EV-D68 neutralizing antibody titers in IVIG products manufactured in the United States and Germany. This study indicated that the IVIG products used in Japan contain neutralizing antibodies against the endemic EV-D68 strains.

In this study, it was the limitation that the neutralization assay was performed once therefore statistical comparisons of IC_50_ remains difficult. Additionally, this study provides only in vitro analysis using RD-A cells for measuring the neutralization activity of IVIG products, therefore further in vivo researches would be necessary for the evaluation of therapeutic treatment.

## Conclusion

In this study, we demonstrated that nine IVIG products used in Japan, including seven products manufactured in Japan and one each manufactured in the United States and Germany, neutralized six domestic EV-D68 strains. Further research will be needed to assess the efficacy of the IVIG products against severe EV-D68 infections in clinical settings.

## Data Availability

The datasets analyzed during the current study are available from the corresponding author on reasonable request.

## List of abbreviations

AFM: Acute flaccid myelitis
AFP: Acute flaccid paralysis
CCID_50_: 50% cell culture infectious dose
CPE: Cytopathic effect
EMEM: Eagle’s Minimum essential medium
EV-D68: Enterovirus D68
FCS: Fetal calf serum
IC_50_: 50% inhibitory concentration
IVIG: Intravenous immune globulin

## References

[1] Baggen J, Thibaut HJ, Strating JRPM, van Kuppeveld FJM (2018). The life cycle of non-polio enteroviruses and how to target it. Nat Rev Microbiol.

[2] Schieble JH, Fox VL, Lennette EH (1967). A probable new human picornavirus associated with respiratory diseases. Am J Epidemiol.

[3] Sun J, Hu XY, Yu XF (2019). Current understanding of human enterovirus D68. Viruses.

[4] Hixon AM, Frost J, Rudy MJ, Messacar K, Clarke P, Tyler KL (2019). Understanding enterovirus D68-induced neurologic disease: a basic science review. Viruses.

[5] Ikeda T, Mizuta K, Abiko C, Aoki Y, Itagaki T, Katsushima F (2012). Acute respiratory infections due to enterovirus 68 in Yamagata, Japan between 2005 and 2010. Microbiol Immunol.

[6] Itagaki T, Aoki Y, Matoba Y, Tanaka S, Ikeda T, Mizuta K, Matsuzaki Y. Clinical characteristics of children infected with enterovirus D68 in an outpatient clinic and the association with bronchial asthma. Infect Dis (Lond) 2018;50:303–312. https://www.tandfonline.com/doi/full/10.1080/23744235.2017.1400176?needAccess=true. 10.1080/23744235.2017.1400176 29119851

[7] Messacar K, Asturias EJ, Hixon AM, Van Leer-Buter CV, Niesters HGM, Tyler KL (2018). Enterovirus D68 and acute flaccid myelitis-evaluating the evidence for causality. Lancet Infect Dis.

[8] Morens DM, Folkers GK, Fauci AS (2019). Acute flaccid myelitis: something old and something new. mBio.

[9] Helfferich J, Knoester M, Van Leer-Buter CCV, Neuteboom RF, Meiners LC, Niesters HG, Brouwer OF (2019). Acute flaccid myelitis and enterovirus D68: Lessons from the past and present. Eur J Pediatr.

[10] Kramer R, Lina B, Shetty J. Acute flaccid myelitis caused by enterovirus D68: Case definitions for use in clinical practice. Eur J Paediatr Neurol 2019;23:235–239. https://www.ejpn-journal.com/article/S1090-3798(18)30281-2/fulltext 10.1016/j.ejpn.2019.01.001 30670331

[11] Mishra N, Ng TFF, Marine RL, Jain K, Ng J, Thakkar R (2019). Antibodies to enteroviruses in cerebrospinal fluid of patients with acute flaccid myelitis. mBio.

[12] Okumura A, Mori H, Fee Chong PF, Kira R, Torisu H, Yasumoto S et al. Serial MRI findings of acute flaccid myelitis during an outbreak of enterovirus D68 infection in Japan. Brain Dev 2019;41:443–451. https://www.brainanddevelopment.com/article/S0387-7604(18)30496-0/fulltext 10.1016/j.braindev.2018.12.001 30594353

[13] Diseases CDC, Division of Viral Diseases, National Centers for Immunization and Respiratory (2015). Division of Vector-borne Diseases, Division of high-consequence Pathogens and Pathology, National Center for emerging and zoonotic infectious Diseases, CDC. Children’s Hospital Colorado; Council of State and Territorial Epidemiologists. Notes from the field: Acute flaccid myelitis among persons aged ≦ 21 years–United States, August 1 - November 13, 2014. MMWR Morb Mortal Wkly Rep.

[14] Aliabadi N, Messacar K, Pastula DM, Robinson CC, Leshem E, Sejvar JJ (2016). Enterovirus D68 infection in children with acute flaccid myelitis, Colorado, USA, 2014. Emerg Infect Dis.

[15] Sejvar JJ, Lopez AS, Cortese MM, Leshem E, Pastula DM, Miller L (2016). Acute flaccid myelitis in the United States, August-December 2014: results of nationwide surveillance. Clin Infect Dis.

[16] Khetsuriani N, Lamonte-Fowlkes A, Oberst S, Pallansch MA (2006). Centers for Disease Control and Prevention. Enterovirus surveillance—United States, 1970–2005. MMWR Surveill Summ.

[17] Holm-Hansen CC, Midgley SE, Fischer TK. Global emergence of enterovirus D68: A systematic review. Lancet Infect Dis 2016;16:e64-e75. https://www.thelancet.com/journals/laninf/article/PIIS1473-3099(15)00543-5/fulltext. 10.1016/S1473-3099(15)00543-5 26929196

[18] Messacar K, Abzug MJ, Dominguez SR. The emergence of enterovirus-D68. Microbiol Spectr. 2016;4. 10.1128/microbiolspec.EI10-0018-2016 10.1128/microbiolspec.EI10-0018-2016 27337448

[19] Pastula DM, Aliabadi N, Haynes AK, Messacar K, Schreiner T, Maloney J (2014). Acute neurologic illness of unknown etiology in children–Colorado, August-September 2014. MMWR Morb Mortal Wkly Rep.

[20] Midgley CM, Jackson MA, Selvarangan R, Turabelidze G, Obringer E, Johnson D (2014). Severe respiratory illness associated with enterovirus D68–Missouri and Illinois, 2014. MMWR Morb Mortal Wkly Rep.

[21] Centers for Disease Control and Prevention. Enterovirus D68. https://www.cdc.gov/non-polio-enterovirus/about/EV-D68.html. Accessed 17 February 2023.

[22] Chong PF, Kira R, Mori H, Okumura A, Torisu H, Yasumoto S (2018). Clinical features of acute flaccid myelitis temporally associated with an Enteerovirus D68 outbreak: results of a nationwide survey of acute flaccid paralysis in Japan, August-December 2015. Clin Infect Dis.

[23] Funakoshi Y, Ito K, Morino S, Kinoshita K, Morikawa Y, Kono T (2019). Enterovirus D68 respiratory infection in a children’s hospital in Japan in 2015. Pediatr Int.

[24] Kaida A, Iritani N, Yamamoto SP, Kanbayashi D, Hirai Y, Togawa M (2017). Distinct genetic clades of enterovirus D68 detected in 2010, 2013, and 2015 in Osaka City, Japan. PLoS ONE.

[25] Ikuse T, Aizawa Y, Yamanaka T, Habuka R, Watanabe K, Otsuka T, Saitoh A (2021). Outbreak of enterovirus D68 among children in Japan-worldwide circulation of enterovirus D68 clade B3 in 2018. Pediatr Infect Dis J.

[26] Zhang Y, Moore DD, Nix WA, Oberste MS, Weldon WC (2015). Neutralization of Enterovirus D68 isolated from the 2014 US outbreak by commercial intravenous immune globulin products. J Clin Virol.

[27] Hixon AM, Clarke P, Tyler KL (2017). Evaluating treatment efficacy in a mouse model of enterovirus D68-associated paralytic myelitis. J Infect Dis.

[28] Rudy MJ, Frost J, Clarke P, Tyler KL (2022). Neutralizing antibody given after paralysis onset reduces the severity of paralysis compared to nonspecific antibody-treated controls in a mouse model of EV-D68-Associated acute flaccid myelitis. Antimicrob Agents Chemother.

[29] Kärber G (1931). Beitrag zur Kollektiven Behandlung pharmakologischer Reihenversuche. Archiv f experiment Pathol u Pharmakol.

[30] Aizawa Y, Watanabe K, Oishi T, Hirano H, Hasegawa I, Saitoh A (2015). Role of maternal antibodies in infants with severe diseases related to human parechovirus type 3. Emerg Infect Dis.

[31] Imamura T, Okamoto M, Nakakita S, Suzuki A, Saito M, Tamaki R (2014). Antigenic and receptor binding properties of enterovirus 68. J Virol.

[32] Vidor E. Poliovirus vaccine-inactivated. In: Plotkin SA, Orenstein W, Offit PA, Edwards KM. Vaccines E-book, 7th edition. Elsevier Health Sciences; 2018. p. 94685–94689 (Kindle version). https://www.elsevier.com/books/plotkins-vaccines/9780323357616

[33] Liu L, Mo Z, Liang Z, Zhang Y, Li R, Ong KC (2015). Immunity and clinical efficacy of an inactivated enterovirus 71 vaccine in healthy chinese children: a report of further observations. BMC Med.

[34] Vogt MR, Crowe JE (2018). Current understanding of humoral immunity to enterovirus D68. J Pediatr Infect Dis Soc.

[35] Schubert RD, Hawes IA, Ramachandran PS, Ramesh A, Crawford ED, Pak JE (2019). Pan-viral serology implicates enteroviruses in acute flaccid myelitis. Nat Med.

[36] Park SW, Pons-Salort M, Messacar K, Cook C, Meyers L, Farrar J, Grenfell BT (2021). Epidemiological dynamics of enterovirus D68 in the United States and implications for acute flaccid myelitis. Sci Transl Med.

[37] Kidd S, Lopez AS, Konopka-Anstadt JL, Nix WA, Routh JA, Oberste MS (2020). Enterovirus D68-associated acute flaccid myelitis, United States, 2020. Emerg Infect Dis.

[38] Carballo CM, Erro MG, Sordelli N, Vazquez G, Mistchenko AS, Cejas C (2019). Acute flaccid myelitis associated with enterovirus D68 in children, Argentina, 2016. Emerg Infect Dis.

[39] Gong L, Wang Y, Zhang W, Chen C, Yang X, Xu L (2020). Acute flaccid myelitis in children in Zhejiang Province, China. Front Neurol.

[40] Pellegrinelli L, Galli C, Primache V, Bubba L, Buttinelli G, Stefanelli P et al. Emerging Non-Polio enteroviruses recognized in the framework of the Acute Flaccid Paralyses (AFP) surveillance system in Northern Italy, 2016–2018. Int J Infect Dis 2021;106:36–40. https://www.ijidonline.com/article/S1201-9712(21)00275-7/fulltext. 10.1016/j.ijid.2021.03.057 33771675

[41] Vogt MR, Fu J, Kose N, Williamson LE, Bombardi R, Setliff I (2020). Human antibodies neutralize enterovirus D68 and protect against infection and paralytic disease. Sci Immunol.

[42] Harrison CJ, Weldon WC, Pahud BA, Jackson MA, Oberste MS, Selvarangan R (2019). Neutralizing antibody against enterovirus D68 in children and adults before 2014 outbreak, Kansas City, Missouri, USA^1^. Emerg Infect Dis.

[43] Karelehto E, Koen G, Benschop K, van der Klis F, Pajkrt D, Wolthers K. Enterovirus D68 serosurvey: Evidence for endemic circulation in the Netherlands, 2006 to 2016. Euro Surveill 2019;24:1800671. https://www.eurosurveillance.org/content/10.2807/1560-7917.ES.2019.24.35.1800671. 10.2807/1560-7917.ES.2019.24.35.1800671 PMC6724466 31481149

[44] Xiang Z, Li L, Ren L, Guo L, Xie Z, Liu C (2017). Seroepidemiology of enterovirus D68 infection in China. Emerg Microbes Infect.

[45] Sun S-Y, Gao F, Hu Y-L, Bian L-L, Mao Q-Y, Wu X. Seroepidemiology of enterovirus D68 infection in infants and children in Jiangsu, China. J Infect 2018;76:563–569. https://www.journalofinfection.com/article/S0163-4453(18)30055-0/fulltext. 10.1016/j.jinf.2018.02.003 29428227

[46] Chan YF, Sam IC, Nayan E, Tan XH, Yogarajah T (2022). Seroepidemiology of enterovirus D68 infection in Kuala Lumpur, Malaysia between 2013 and 2015. J Med Virol.

[47] Lee JT, Shih WL, Yen TY, Cheng AL, Lu CY, Chang LY, Huang LM (2020). Enterovirus D68 seroepidemiology in Taiwan, a cross sectional study from 2017. PLoS ONE.

